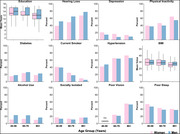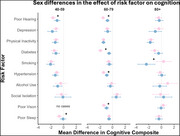# The sex divide: Modifiable risk factors and their relative associations with cognition

**DOI:** 10.1002/alz70860_102110

**Published:** 2025-12-23

**Authors:** Megan Fitzhugh, Judy Pa

**Affiliations:** ^1^ University of California, San Diego, San Diego, CA, USA; ^2^ University of California San Diego, La Jolla, CA, USA; ^3^ Alzheimer's Disease Cooperative Study, La Jolla, CA, USA

## Abstract

**Background:**

Women have a greater lifetime risk of developing Alzheimer's disease (AD) and make up approximately two‐thirds of AD cases. Despite these clear sex differences, most studies investigating risk factors for the disease only include sex as a covariate. This study was a comprehensive analysis of sex differences in both the prevalence of modifiable risk factors of AD and their association with cognition.

**Method:**

This study included 17,182 participants aged 40+ from the Health and Retirement Study (69.2±10.6 years, 59.2% female). Participants were stratified by age into 3 groups, 40‐59 years, 60‐79 years, and 80+ years. The presence of twelve modifiable risk factors (Yes/No) were ascertained from medical records and self‐report questionnaires. A cognitive composite score derived from the TICS was the outcome measure. Chi‐square was used to examine sex differences in the prevalence of risk factors, and logistic regression was used to examine sex by age group differences in the prevalence of risk factors. ANOVAs were used to investigate the three‐way interaction of sex, age group, and presence of risk factor on cognition.

**Result:**

There were significant sex differences, with women bearing more risk factors than men (Figure 1). More men experienced hearing loss and alcohol abuse, while more women experienced depression, physical inactivity, diabetes, smoking, social isolation, vision impairment, poor sleep, and lower education. The effect of risk factors on cognition also differed by sex and age group (Figure 2). Women with hearing loss, diabetes, hypertension, vision impairment, and poor sleep had worse cognition than those without the risk factor. Men who smoked had worse cognition than those who did not.

**Conclusion:**

This study examined sex differences in multiple modifiable risk factors of AD and their relationship with cognition. We found that women experienced several more risk factors compared to men. Importantly, the risk factors had a larger, negative effect on cognition in women compared to men with the same risk factor. Together, these data suggest that women may be at greater risk of AD because they carry more risk factors, which have a greater impact on cognition. Results may inform future personalized prevention strategies for dementia risk reduction.